# Atomic structure of the Epstein-Barr virus portal

**DOI:** 10.1038/s41467-019-11706-8

**Published:** 2019-08-29

**Authors:** Cristina Machón, Montserrat Fàbrega-Ferrer, Daming Zhou, Ana Cuervo, José L. Carrascosa, David I. Stuart, Miquel Coll

**Affiliations:** 1 Institute for Research in Biomedicine (IRB Barcelona), The Barcelona Institute of Science and Technology (BIST), Baldiri Reixac 10-12, 08028 Barcelona, Spain; 2 Molecular Biology Institute of Barcelona (IBMB-CSIC), Baldiri Reixac 10-12, 08028 Barcelona, Spain; 3 Division of Structural Biology, University of Oxford, The Henry Wellcome Building for Genomic Medicine, Headington, Oxford, OX3 7BN UK; 4 Department of Macromolecular Structures, Centro Nacional de Biotecnología-Consejo Superior de Investigaciones Científicas (CNB-CSIC), 28049 Madrid, Spain

**Keywords:** Herpes virus, Cryoelectron microscopy

## Abstract

*Herpesviridae* is a vast family of enveloped DNA viruses that includes eight distinct human pathogens, responsible for diseases that range from almost asymptomatic to severe and life-threatening. Epstein-Barr virus infects B-cells and epithelial cells, causing infectious mononucleosis, as well as a number of cancers. Epstein-Barr infection cannot be cured since neither vaccine nor antiviral drug treatments are available. All herpesviruses contain a linear double-stranded DNA genome, enclosed within an icosahedral capsid. Viral portal protein plays a key role in the procapsid assembly and DNA packaging. The portal is the entrance and exit pore for the viral genome, making it an attractive pharmacological target for the development of new antivirals. Here we present the atomic structure of the portal protein of Epstein-Barr virus, solved by cryo-electron microscopy at 3.5 Å resolution. The detailed architecture of this protein suggests that it plays a functional role in DNA retention during packaging.

## Abstract (web summary)

The Epstein-Barr virus (EBV) is a dangerous human pathogen responsible for mononucleosis and several types of cancers. Here the authors describe a high-resolution atomic structure of the EBV portal, which serves as the entrance and exit pore for the viral genome and is a potential pharmacological target for the development of antivirals.

## Introduction

The eight herpesviruses infecting humans cause different disorders and are classified into three subfamilies, namely *α*, *β*, and *γ*. Alphaherpesvirinae include herpes simplex virus types 1 and 2 (HSV-1, HSV-2), and varicella-zoster virus (VZV), causing herpes labialis/genitalis and chickenpox, respectively. Betaherpesvirinae include cytomegalovirus (CMV) and roseolovirus (HHV-6, HHV-7), which cause infectious mononucleosis and roseola infantum, respectively. Finally, gammaherpesvirinae, which include Kaposi’s sarcoma-associated virus (KSHV) and Epstein-Barr virus (EBV) are associated with different types of cancer. EBV causes infectious mononucleosis, a number of cancers, such as Burkitt’s, Hodgkin’s and T-cells lymphomas, and gastric and naseopharyngeal carcinomas and several autoimmune diseases such as lupus erythematosus^1–3^.

All herpesviruses have a portal pore for capsid assembly and DNA packaging and ejection. On the basis of their capsid structure and viral DNA packaging mechanism, it has been suggested that herpesviruses and tailed bacteriophages are evolutionarily related^4–6^. Portal proteins are located at a unique capsid vertex and are one of the conserved elements within herpesvirus and bacteriophages, even though there is no sequence similarity between them, nor among the different bacteriophage portals solved. In early stages of infection, portal proteins are involved in the release of the viral genome from the capsid to the cell. Transcription of the viral genes, together with extensive replication of long concatameric DNA, takes place once a lytic infection is triggered. Later in the infection, portal proteins are among the first proteins recruited during the assembly of the new procapsids. In addition, they interact with the terminase complex, which provides the energy and force necessary for packaging the DNA inside the capsid and cleaves the DNA at the appropriate site. In bacteriophages, portal proteins also serve as a connection between the capsid and the tail proteins^7,8^. Given their key function in DNA packaging during viral replication^9^, portal proteins are attractive targets for the development of antiviral drugs. However, high-resolution atomic structures are not available for structure-driven drug design that specifically targets the portal.

The structure determination at high resolution of portal proteins from bacteriophages has been successful with examples of gp6 from SPP1, gp1 from P22, gp20 from T4, and gp10 from ϕ29, solved by X-ray crystallography and cryo-EM^10–14^. However, the study of herpesvirus portals has been more challenging because of the difficulty in expressing them in soluble form and in obtaining crystals. This difficulty is likely attributable to a large flexible area in their structure (see below). Consequently, to date, only limited structural studies of herpesvirus portals have been performed, all at low resolution^15–18^. While we were submitting this paper, a cryo-EM structure of the portal vertex of HSV-1, an alpha herpesvirus, was published. The structure was solved by symmetry relaxation of the whole virion, with the portal determined at 5.6 Å resolution, although coordinates and maps were not yet available for precise comparison with our EBV portal^19^. Here we present the atomic structure of the EBV portal solved by cryo-electron microscopy at 3.5 Å resolution, showing its detailed architectural features.

## Results

The putative portal protein of EBV, pBBRF1, which has an expected molecular weight of 68 kDa as a monomer, was expressed in insect cells and purified as a fusion protein with a His-Z-tag in four chromatography steps, in the presence of n-dodecyl β-D-maltoside to increase its stability. The quality and homogeneity of the sample was tested by negative-stain electron microscopy (data not shown) and purified protein was used for single-particle cryo-EM (Supplementary Fig. 1), and two different datasets were collected from different preparations. The data rendered density maps at 3.5 and 3.6 Å resolution, which were of sufficient quality for the de novo building and refinement of an atomic model of the protein (Supplementary Fig. 1 and Supplementary Table 1).

**Fig. 1:**
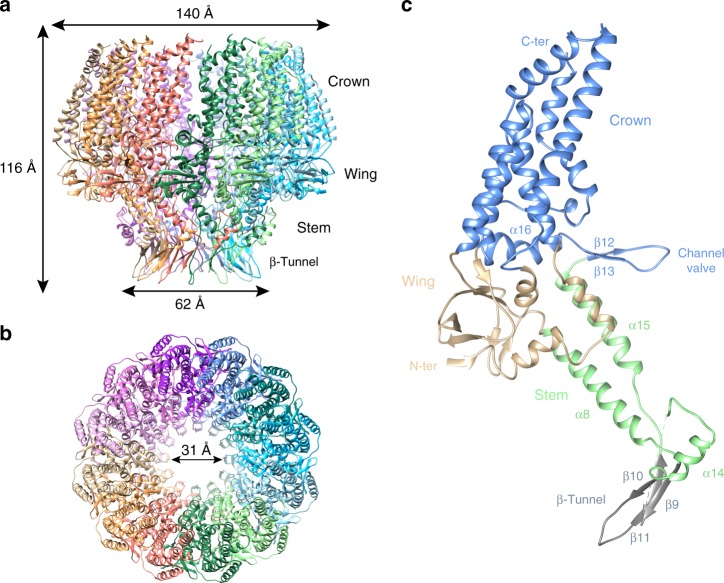
Structural characterization of EBV portal. Ribbon representation of the EBV portal cryo-EM structure, indicating the dimensions of the particle, and diameter of the inner channel. **a** Lateral view. **b** Axial view. **c** Ribbon representation of EBV portal monomer colored by regions: crown (blue), wing (sand), stem (light green), and β-hairpin channel valve (gray). The channel valve and secondary elements relevant for protein–protein interactions are also indicated

**Fig. 2:**
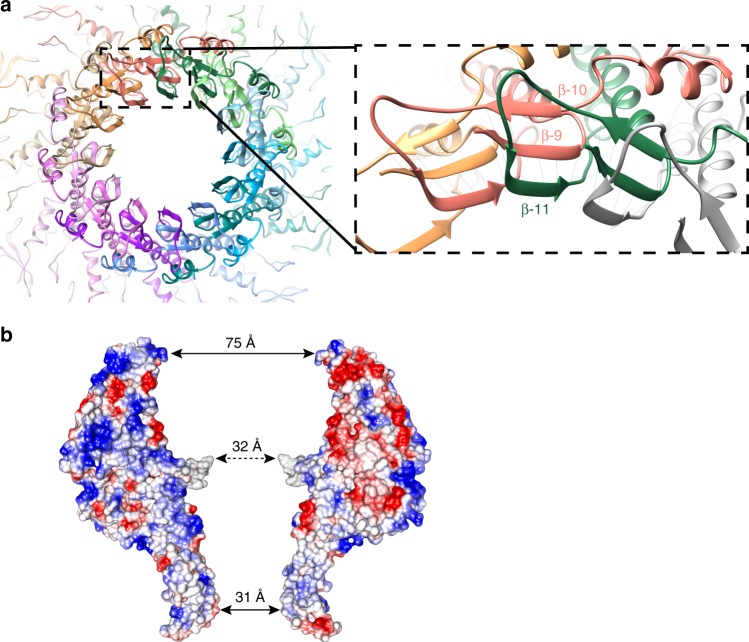
Protein–protein interactions in the EBV portal oligomer. **a** Axial view of EBV portal protein at the β-tunnel region (left), and a close-up view of protomer–protomer β-sheet formation (right) with β-strands 9, 10 of one protomer and 11, from an adjacent protomer. **b** Electrostatic potential of two opposing monomers within EBV portal dodecamer. Blue represents 10 kcal/(mol · *e*) positive potential, while red represents −10 kcal/(mol · *e*) negative potential. Indication of the distance between residues in the central part of the channel at different levels is included. Gray shows the six residues of the tunnel loop with weak density

The EBV portal shows a ring-like structure, consisting of 12 subunits. In contrast to bacteriophage portals, where particles have been reported as 12-mers and 13-mers^10–14^, no indication of an oligomerization state other than 12 was detected in the micrographs or 2D averages. The particle of the EBV portal has a mushroom-like shape, with a maximum external diameter of 140 Å at the “cap” and 62 Å at the “stem”, while the height is 116 Å (Fig. 1). There is an internal conical channel which narrows from the “cap” of the mushroom to its “stem”, with a diameter ranging from 75 to 31 Å, respectively (Fig. 2). The wide “cap” points to the interior of the capsid, while the narrow “stem” makes the channel to the exterior (see below). The structure comprises three domains. There are marked differences from the bacteriophage portals, however, for comparison purposes we follow their nomenclature and name the domains wing, stem, and crown (Fig. 1, Supplementary Movie 1). The wing or central domain connects the crown to the stem, and contains eight β-strands—three of them relatively short—and three short α-helices (Fig. 1). There are two nearly perpendicular β-sheets, each of them composed of three antiparallel β-strands, forming an SH3-like structure, similar to that found in portal protein gp10 of ϕ29^10^. The N-terminus is also located in this domain, although the first 16 residues are not resolved in our maps. The wing is the outermost domain, however, the EBV portal wing protrudes less than the corresponding structure in bacteriophage portals, since the wide part of the crown cone has about the same external diameter. The stem region comprises two antiparallel α-helices with different length, α8 and α15 (Fig. 1), which shape the walls of the channel, a feature conserved among all portal proteins. They are tilted relative to the tunnel axis by 45° and 30°, respectively. After the two helices, there are three β-strands and a further short helix (α14) that embraces the neighboring monomer and is positioned almost perpendicular to α8 (Fig. 1). After the β-strands there are five predicted helices that are not assigned in the current structure (from residue 288 to 433) because the density is too poor to allocate their position, which reflects the flexibility of the distal tip region of the EBV portal. Based on the weak density in this area, we can, however, infer that these helices make a longer channel after the β-tunnel, and are flexible in order to adjust to the fivefold symmetry of the portal vertex protein (see below).

### The channel valve of EBV portal

The crown is the most variable region in bacteriophage portal proteins. This region is absent in phage ϕ29 portal^10,14^, while in P22 portal it has a long α-barrel shaped structure (Supplementary Fig. 2)^12^. In the EBV portal, the crown is larger than in most bacteriophage portals and includes at least eight α-helices of varying size. There is a clear separation between the wing and the crown with no structured secondary structure elements crossing between the two domains (except for the channel valve, see below), however, the deep cleft between the domains observed in some bacteriophage portals, is not present (Fig. 1a–c and S2). The C-terminus of the protein should be located at the crown, although the density of the last 40 residues is not clearly visible. In the monomer, the crown domain is tilted about 66° relative to the stem domain, with the wing acting as a hinge (Fig. 1c). The channel valve^20^ (also termed tunnel loop) is located at the crown and the wing interface, at the channel interior. In most bacteriophage portals, the channel valve is formed by a long α-helix which protrudes from the wing towards the channel and ends in the tunnel loop, which is between 10 and 26 residues long^11–13^ and connects back to the stem. This helix and loop have been proposed to undergo a movement that plays a crucial role during DNA packaging^11,20^. However, in the EBV portal, the equivalent helix, α16, is shorter, forms part of the crown, not of the wing, and does not protrude into the channel. Instead, α16 continues in the channel as a β-strand that forms a β-ribbon, with the tunnel loop at its tip. The β-ribbon valve thus connects α16 with α15, the latter being one of the two long helices of the stem. This loop of the β-ribbon is not well defined, showing weak density for the six residues at the tip, indicating the flexibility of this region, a feature also observed in bacteriophage portals. Without these six residues, the aperture inside the channel at this level would be around 36 Å. However, when residues are modeled into the weak density, the aperture is reduced to about the diameter of the B-form DNA. This structural feature clearly corresponds to the valve observed in bacteriophage portal structures^20^ and most probably plays a similar role in securing the DNA inside the capsid. It is interesting to note the position of helix α16, which is oriented perpendicular to the channel axis and between monomers. Inter-monomer contacts at the crown are scarce, giving the impression that there is a cleft between the protomers (Fig. 1a, Supplementary Fig. 1, and Supplementary Movie 1).

### A β-tunnel forms a second narrow section

In the EBV portal, in addition to the channel valve, another narrow section in the channel is located at end of the stem, at the level of strand β11 (Fig. 2). This leaves an aperture of 31 Å, which would allow easy passage of B-DNA. This constriction is quite different from the channel valve, since it extends for about 17 Å along the channel, forming a tunnel. Furthermore, it is a fixed structure with no indication of mobility. Indeed, this cylindrical structure is formed by the inter-monomer assembly of 12 three-stranded β-sheets with their planes radially disposed around the channel in the dodecamer (Fig. 2a). Two β-strands—parallel to each other—belong to one monomer, while the third β-strand, antiparallel, belongs to the adjacent subunit, thus building a strong interaction between monomers. A similar arrangement has been found in bacteriophage portal proteins^10,11,13^, however, the EBV portal differs in the disposition of these β-sheets, since their edges face the interior of the channel, forming the stiff cylindrical structure that we name the β-tunnel (Fig. 2). This structure, which includes the hydrophobic side chain of Phe470 at the channel surface, is expected to play a role in guiding DNA passage, but there is no indication for it being a gate or a valve with an open/close motion.

Adjacent protomers of the EBV portal dodecamer have a large contact area of 4050 Å^2^. The oligomer is stabilized by a complex network of interactions, located mostly at the stem domain, where each protomer interacts with up to four distinct neighboring protomers. In addition to the inter-protomer interactions involved in the assembly of the β-tunnel described above, helix α8 also contributes to dodecamer stabilization. The residues of this helix interact with three distinct monomers, in such a way that residues of helix α8 of monomer A interact with residues in the loop before helix α14 of monomers B and C, as well as residues of helix α8 of monomer L. There are also some complementary charged areas buried in the interacting surface between monomers in the wing and crown regions, which establish salt bridges between them (Fig. 2b). A putative disulfide bridge between Cys166 and Cys254 has been described in pUL6 from HSV-1. This may contribute to the stabilization of HSV-1 portal, since the presence of dithiotreitol results in the disruption of the dodecameric rings, and the mutation of those residues produced capsids with a reduced level of pUL6^21^. These Cys residues are not conserved in EBV and we did not find any other disulfide bridge in the current structure.

### Interaction of EBV portal with capsid proteins

**Fig. 3:**
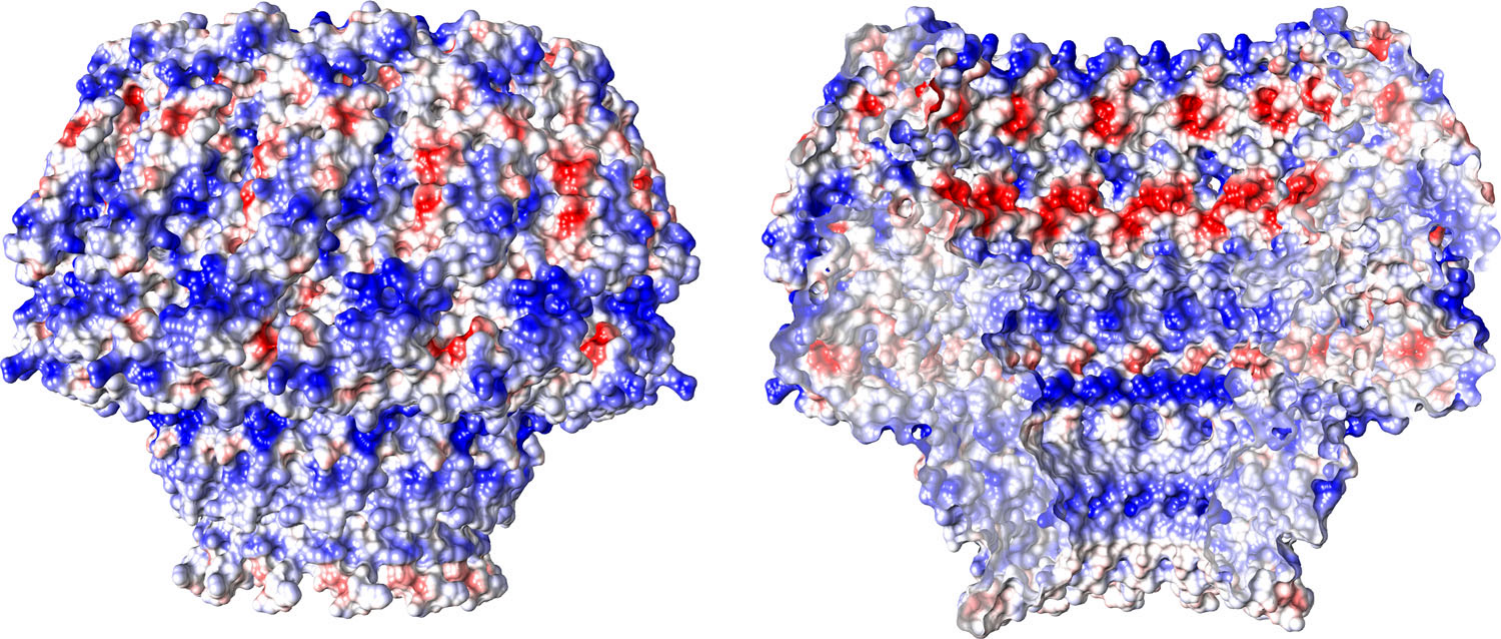
EBV portal electrostatic potential. **a** Electrostatic potential on the external (left) and inner (right) surfaces of EBV portal dodecamer. Blue represents 10 kcal/(mol · *e*) positive potential, while red represents −10 kcal/(mol · *e*) negative potential

**Fig. 4:**
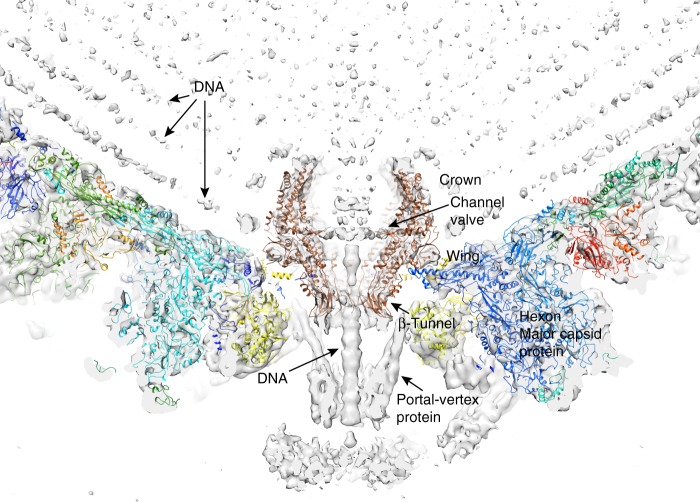
EBV portal fitting in the HSV-1 capsid portal vertex map. The EBV portal structure (brown-sienna, this work) and the capsid components of HSV-2 (rainbow colors, PDB 5ZZ8^22, 24^) were docked into the portal vertex density of HSV-1 capsid, at the unique portal vertex (gray, EMD 4347^23^) with Chimera^44^. The relevant domains of EBV portal protein, the interacting capsid proteins, as well as the viral DNA are indicated

The electrostatic potential of the EBV portal is distinct from that of the bacteriophage portal proteins (Fig. 3), the external surface of the EBV portal is mainly positively charged, while the phage portals are mostly negative^13^. This difference may have implications for the interactions established between the EBV portal and the rest of the proteins of the capsid and the packaged DNA. Figure 4 shows our EBV portal structure fitted in the portal vertex of the HSV-1 8 Å density with the atomic coordinates of the HSV-2 capsid also fitted (with a penton of the major capsid protein removed at the pentameric vertex to accommodate the portal)^22–24^. The good fit of the portal in the density, with a correlation coefficient of 0.7803—even though the density was fivefold averaged instead of 12-fold as for the portal protein—indicates that the position of the portal with respect to the capsid is correct. The figure clearly indicates that the portal is rather internal, with its crown fully inside the capsid. The portal is held mainly through its wing, which interacts with the major capsid protein of the adjacent hexons, and also with an unknown helical pentameric protein at the end of the so-called clip in the bacteriophage portals. It is interesting that the wing has a SH3-like domain, a structure which mediates protein–protein interactions in other systems^25^. The three positively charged rings of the crown suggest that they play a role in the interaction with the packaged DNA phosphates (Fig. 3), since they roughly coincide with layers of the DNA genome. The density of the portal vertex reveals that the β-ribbon channel valve is well ordered and closed, with the internal DNA pressed against the valve. An open question is whether the valve closes due to a movement of the crown, as a result of the pressure of the DNA once the capsid is full—a movement that could be transmitted to α16 and its contiguous β-ribbon to close the valve. The loose interaction between protomers at the crown indicates that the crown might be compressible.

This work describes a high-resolution atomic structure of a herpesvirus portal particle. An alignment of EBV portal sequence against the sequences of all the other portal proteins of herpesviruses shows 23–25% sequence identity, except for pORF43 from Kaposi’s sarcoma-associated herpesvirus, which shows 51% sequence identity, since it belongs to the same subfamily of gammaherpesvirinae. Among the most conserved regions between all herpesvirus portals are those involved in protein–protein interactions, such as α8 helix or β11 strands, and the channel valve/tunnel loop region.

The drugs currently licensed for the treatment of herpesvirus infections target the viral DNA polymerase. They show poor efficacy because of the appearance of resistant viral strains or serious side effects after a prolonged treatment^26–28^. In the case of EBV infection, the situation is even more critical, because there is no specific treatment for this severe disease. Given the key role of portal proteins in herpesvirus DNA packaging and release, as well as during capsid formation, their atomic structures will be extremely valuable for the rational design of compounds targeting their function. A number of herpes portal inhibitors have been reported^9^; however, their modes of action and binding are unknown, hindering their further development. The structure presented here paves the way to prepare and solve inhibitor-portal binary complexes, which in turn will accelerate their optimization and allow the design of new highly specific antivirals.

## Methods

### Cloning of *bbrf1* gene

The *bbrf1* gene, which codes for the EBV portal protein, was amplified by PCR from genomic DNA purchased from The National Collection of Pathogenic Viruses (NCPV) (Epstein-Barr virus #634), using the oligonucleotides (the sequence annealing with *bbrf1* is indicated in bold) bbrf1_EBV_pPEU10_F: 5′-AAGTTCTGTTTCAGGGCCCG**ATGTTCAACATGAACGTGGACGA**-3′ and bbrf1_EBV_pPEU10_R: 5′-ATGGTCTAGAAAGCTTTA**ACTTCCAGCACCAGGCGGG**-3′. The 1842-bp PCR-amplified fragment was cloned in *Kpn*I-*Hind*III digested pPEU11 vector (provided by the Protein Expression Facility, at the Institute for Research in Biomedicine (IRB-Barcelona)) by an In-Fusion® reaction (Clontech). The resulting construct, pCMS1017, was checked by sequencing and used for the expression of the fusion protein His-Z-pBBRF1 in Baculovirus.

### Purification of pBBRF1 from recombinant baculovirus

Recombinant baculovirus was produced following the instructions of baculoCOMPLETE^TM^ ALL-IN-ONE Baculovirus Protein Expression kit from Oxford Expression Technologies. The protein expression conditions were optimized by infection of a monolayer of *Spodoptera frugiperda* Sf9 cells with the produced baculovirus. The cells were harvested after 72 h of incubation at 28 °C, when the expression of His-Z-pBBRF1 was optimal, by centrifugation of the media at 3000 × *g* for 20 min at 4 °C. The cell pellet was lysed by pipetting in lysis buffer (20 mM Tris-HCl pH 8.0, 500 mM NaCl, 20 mM imidazole, 5 mM β-mercaptoethanol, and 0.2% n-dodecyl-beta-maltoside (DDM) (w/v)), and passing it through a cell disruptor (Constant Systems Ltd) at 25 kpsi. Following centrifugation at 40,000 × *g* for 20 min at 4 °C, the sample was filtered and loaded on a HisTrap^TM^ HP 5-ml (GE Healthcare Life Sciences). His-Z-pBBRF1 was eluted from the column with a gradient between 20 and 500 mM imidazole (20 mM Tris-HCl pH 8.0, 500 mM NaCl, 5 mM β-mercaptoethanol, and 0.05% n-dodecyl-beta-maltoside (DDM) (w/v)). The fractions containing His-Z-pBBRF1 were pooled and loaded on a HiTrap^TM^ Heparin HP 1-ml (GE Healthcare Life Sciences) and eluted with a gradient between 150 mM and 1.5 M NaCl of heparin buffer (50 mM Tris-HCl pH 8.0, 20 mM β-mercaptoethanol, 0.05% DDM (w/v), 1 mM EDTA). Fractions enriched with His-Z-pBBRF1 were then loaded on a HiPrep 16/60 Sephacryl S-400 HR (GE Healthcare Life Sciences), equilibrated with heparin buffer with 500 mM NaCl, and afterwards on a Superose 6 10/300 GL (GE Healthcare Life Sciences). The eluted fractions were analyzed by gel electrophoresis and concentrated with an Amicon Ultra 100K. At this stage, the protein was ready for further analysis and characterization.

### Cryo-EM data collection

Cryo-EM samples were prepared in Quantifoil R2/2 copper grids coated with a thin carbon film, which was made in house. The grids were glow discharged for 60 s at 25 mA using an Emitech Glow Discharge machine, before applying 3 µl of purified His-Z-pBBRF1 at a concentration of 0.1 mg/ml. The sample was incubated for 2 min at room temperature on the grid, before being blotted and applying another 3 µl of heparin buffer with 500 mM NaCl, without DDM. This procedure was repeated twice, before the final blotting of the sample on a VitroBot Mark IV (FEI) system at 20 °C and 95% humidity, for 3.5 s, blotting force: 0, and plunge-freezing it into liquid ethane.

Data were collected on a Titan Krios (FEI) transmission electron microscope at 300 keV, using a Gatan Quantum energy filter and a K2 Summit direct detector, at the Electron Bio-Imaging Centre (eBIC) at Diamond Light Source (Oxford, UK) and The Netherlands Centre for Electron Nanoscopy (NeCEN) at the Universiteit Leiden (Leiden, The Netherlands). In total, 2172 and 5431 movies were collected at eBIC and at NeCEN, at a 1.06 and 1.1 Å pixel size, respectively, within a defocus range from −1.0 to −3.0 µm and at a dose rate of 6.7 e^−^/Å^2^/s, in Super-Resolution mode for eBIC.

### Cryo-EM images processing and map calculation

Cryo-EM data processing was performed using both the Scipion software framework^29^ and RELION 3.0^30^. Dose-fractionated image stacks were motion-corrected and dose-weighted using MotionCor2^31^. Contrast transfer function (CTF) was estimated using the CTFfind4 program^32^. Particles were picked using xmipp3^33^, RELION auto-picking, and Gautomatch within the Scipion software framework^29^. Extracted particles were classified using RELION 2D and 3D, after building an initial volume with Ransac^33^ (NeCEN data) or RELION (eBIC data), applying C12 symmetry to the model. Final volumes were obtained using RELION Auto-refine with 73,395 particles for the model at 3.5 Å resolution (NeCEN data) and with 25,788 particles for the model at 3.6 Å (eBIC data). The structure resolution was estimated from RELION FSC curves with the 0.143 cutoff criterion, and local resolution was computed with MonoRes^34^.

### Structure solution, model building, and coordinate refinement

A partial model of EBV portal was obtained after providing the sequence of pBBRF1 and the cryo-EM map to Buccaneer^35^, within CCP-EM framework^36,37^. The remaining structure was built ab initio in Coot^38^, considering the secondary structure prediction obtained from PSIPRED^39,40^ as a guide. PHENIX real space refinement^41^ and REFMAC5^42^ within the CCP-EM suite for cryo-EM data were used to refine the models. The models were validated using MolProbity^43^. Both models are very similar, with an r.m.s.d. of 0.88 Å, although the eBIC data map was better defined in some peripheral areas, and helix α6 could be assigned in this case. Clear density, although weak, was also present at the loop of the β-hairpin tunnel valve in this map.

### Reporting summary

Further information on research design is available in the Nature Research Reporting Summary linked to this article.

## Supplementary information

Supplementary Information

Reporting Summary

Description of Additional Supplementary Files

Supplementary Movie 1

## Data Availability

The electron microscopy maps have been deposited in the Electron Microscopy Data Bank (EMDB) and Protein Data Bank with accession codes EMD-10010 and EMD-10011, 6RVR, and 6RVS, respectively. All other relevant data are available from the authors upon request.
