# Supplementary material for: Atomic structure of the Epstein-Barr virus portal
Source: Nat Commun. 2019 Aug 29;10:3891. doi: 10.1038/s41467-019-11706-8 (PMC6715670; doi:10.1038/s41467-019-11706-8)
Supplement: Supplementary file 1 — Supplementary Information [file 41467_2019_11706_MOESM1_ESM.pdf]

SUPPLEMETARY INFORMATION

Atomic structure of the Epstein-Barr virus portal

Machón et al.

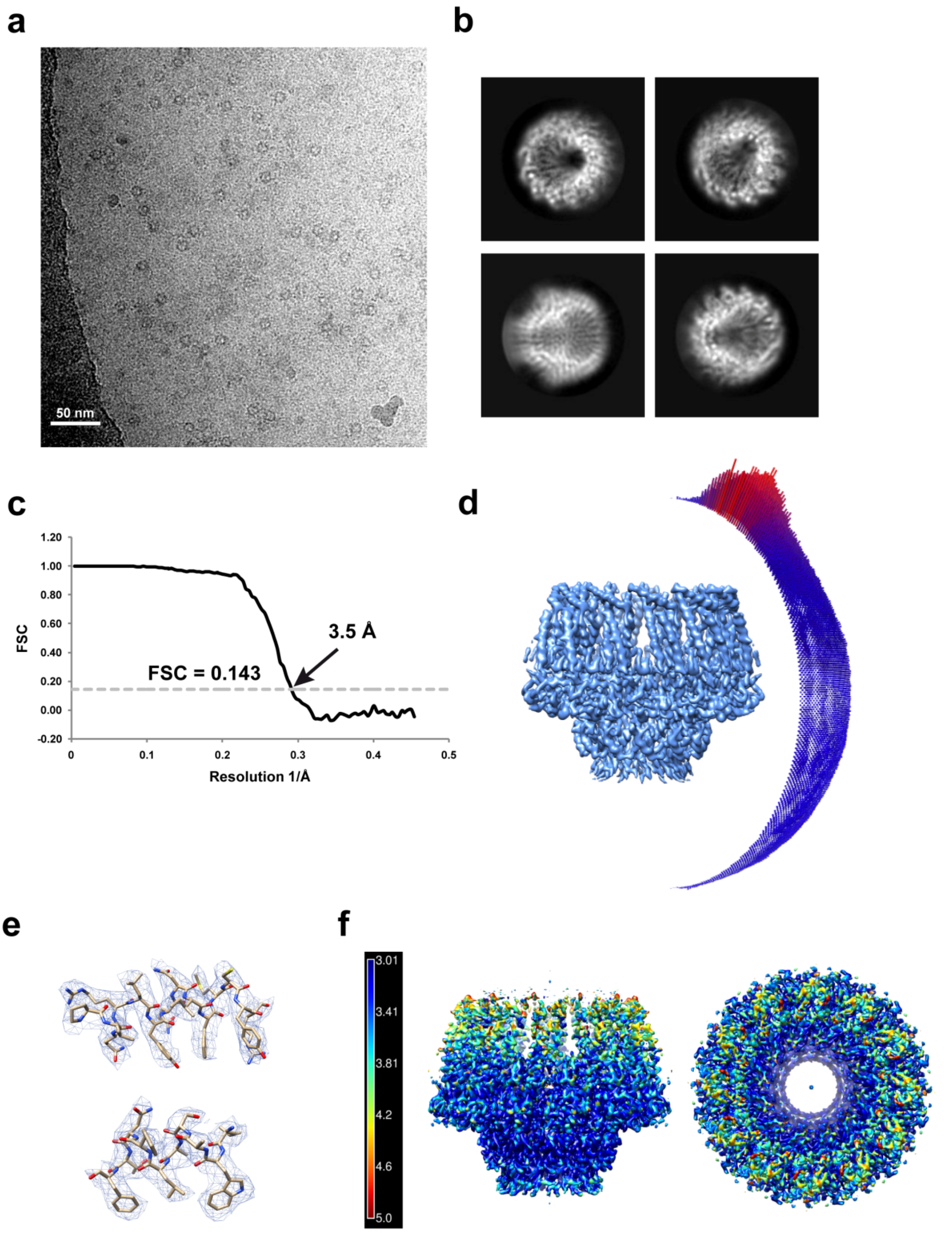

Supplementary Fig. 1. a. Representative cryo-EM micrograph of the EBV portal sample. The scale bar represents 50 nm. **b.** RELION 2D averages used for model building, showing end-on and side-views. **c.** RELION resolution FSC curve. The FSC= 0.143 gold-standard cutoff criterion was used for determining the final resolution of the model (3.5 Å). **d.** RELION 3D reconstruction of EBV portal dodecamer model showing the angular distribution plot of the particles. **e.** Extraction of the density from the 3D reconstruction showing the placement of the side chains in two different areas. **f.** MonoRes local resolution map showing a side view (left) and an end-on view (right). The resolution plot ranges from 3.01-5.0 Å. All panels were generated from the data set collected at NeCEN.

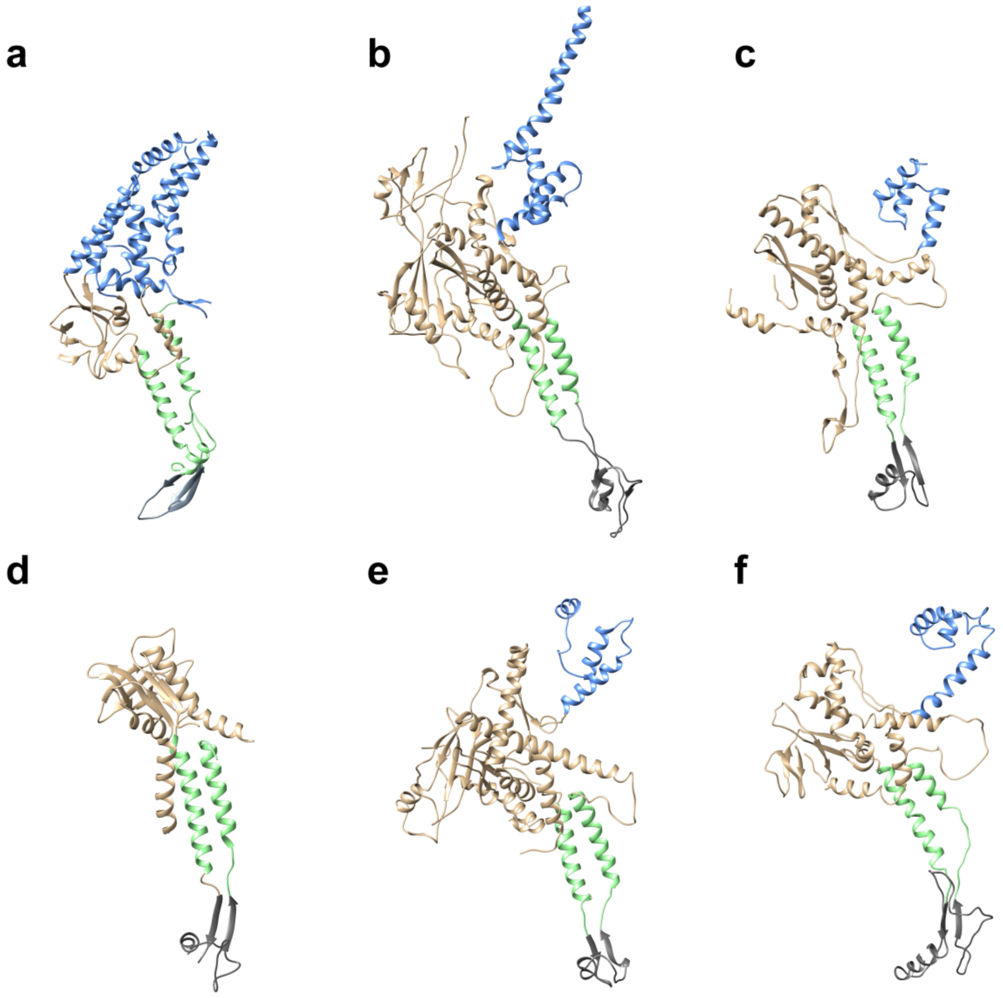

Supplementary Fig. 2. Comparison of EBV portal with bacteriophage portal proteins. Portal proteins in monomeric display with the wing, crown, stem and clip domains colored in sand, blue, light green and gray, respectively. **a.** EBV portal protein. **b.** gp1 P22 portal protein (3LJ5). **c.** gp6 SPP1 portal protein (2JES). **d.** gp10 ϕ29 portal protein (1H5W). **e.** gp8 T7 portal protein (6QX5). **f.** gp20 T4 portal protein (3JA7).

Supplementary Table 1. Cryo-EM data collection, refinement and validation statistics.

	**His-Z-pBBRF1 NeCEN EMD-10010 PDB 6RVR**	**His-Z-pBBRF1 DLS EMD-10011 PDB 6RVS**
**Data collection and processing**		
Nominal magnification	130,000	130,000
Voltage (kV)	300 kV	300 kV
Electron exposure (e^-/Å^2)	40	40
Defocus range (μm)	- 1.0 to - 3.0	- 1.0 to - 3.0
Pixel size (Å/pix)	1.10	1.06
Symmetry imposed	C12	C12
Initial particle images (no.)	404,914	250,788
Final particle images (no.)	73,395	35,063
Map resolution (Å)	0.143	0.143
FSC threshold	3.5 Å	3.6 Å
Map resolution range	3.5 to 5 Å	3.6 to 5 Å
**Refinement**		
Initial model used	None (*ab-initio*)	3.46 Å resolution model
Map sharpening *B* factor (Å^2)	-189.91	-165.00
Model composition		
Non-hydrogen atoms	37668	38340
Protein residues	4680	4764
B factors (Å^2)		
Protein	28.20	81.18
R.m.s. deviations		
Bond lengths (Å)	0.01	0.01
Bond angles (°)	1.137	0.971
Validation		
MolProbity score	2.34	1.76
Clashscore	9.44	4.62
Poor rotamers (%)	2.87	0.82
Ramachandran plot		
Favored (%)	91.71	91.00
Outliers (%)	0.26	0.51
