# Supplementary material for: Atomic structure of the Epstein-Barr virus portal
Source: Nat Commun. 2019 Aug 29;10:3891. doi: 10.1038/s41467-019-11706-8 (PMC6715670; doi:10.1038/s41467-019-11706-8)
Supplement: Supplementary file 3 — Description of Additional Supplementary Files [file 41467_2019_11706_MOESM3_ESM.docx]

**Description of Additional Supplementary Files**

File Name: Supplementary Movie 1
Description: EBV portal protein. Lateral and axial views rotation of EBV portal protein, with close up view of the channel valve, with the wing, crown, stem and β-tunnel domains colored in sand, blue, light green and gray, respectively.
